# T Helper 17-Associated Cytokines Are Produced during Antigen-Specific Inflammation in the Mammary Gland

**DOI:** 10.1371/journal.pone.0063471

**Published:** 2013-05-16

**Authors:** Pascal Rainard, Patricia Cunha, Salim Bougarn, Angélina Fromageau, Christelle Rossignol, Florence B. Gilbert, Patricia Berthon

**Affiliations:** 1 Infectiology and Public Health Research Unit, Institut National de la Recherche Agronomique, Nouzilly, France; 2 Infectiology and Public Health Research Unit, Université François Rabelais de Tours, Nouzilly, France; NIAID, United States of America

## Abstract

Infectious mastitis cuts down milk production profitability and is a major animal welfare problem. Bacteria-induced inflammation in the mammary gland (MG) is driven by innate immunity, but adaptive immunity can modulate the innate response. Several studies have shown that it is possible to elicit inflammation in the MG by sensitization to an antigen subsequently infused into the lumen of the gland. The objective of our study was to characterize the inflammation triggered in the MG of cows sensitized to ovalbumin, by identifying the cytokines and chemokines likely to play a part in the reaction. Among immunized cows, responders mobilized locally high numbers of leukocytes. An overexpression of the genes encoding IL-17a, IL-17F, IL-21, IL-22 and INF-γ was found in milk cell RNA extracts in the early phase of the inflammatory response. At the protein level, IL-17A was detected in milk as soon as the first sampling time (8 h post-challenge), and both IL-17A and IFN-γ concentrations peaked at 12 to 24 h post-challenge. In mammary tissue from challenged quarters, overexpression of the genes encoding IL-17A, IL-17F, IL-21, IL-22, IL-26 and IFN-γ was observed. Neutrophil-attracting chemokines (CXCL3 and CXCL8) were found in milk, and overexpressed transcripts of chemokines attracting lymphocytes and other mononuclear leukocytes (CXCL10, CCL2, CCL5, CCL20) were detected in mammary tissue. Expression of IL-17A, as revealed by immunohistochemistry, was located in epithelial cells, in leukocytes in the connective tissue and in association with the epithelium, and in migrated alveolar leukocytes of challenged quarters. Altogether, these results show that antigen-specific inflammation in the MG was characterized by the production of IL-17 and IFN-γ. The orientation of the inflammatory response induced by the antigen-specific response has the potential to strongly impact the outcome of bacterial infections of the MG.

## Introduction

Bacterial infections of the mammary gland (MG) pose a major problem to the dairy industry worldwide by jeopardizing the profitability of dairy farming. They also constitute the major reason for the use of antibiotic therapy in cattle. Among the different possible approaches to reducing the mastitis problem, the modulation of MG immune defenses is attracting a lot of interest. The response of the MG to infection is characterized by a neutrophilic inflammation. Mastitis induces milk leukocytosis, polymorphonuclear neutrophils being the dominant cell type in milk in acute and chronic mastitis [1]. Neutrophils play a key role in the defense of the MG, and their prompt mobilization from blood into milk is crucial to prevent the proliferation of fast-growing bacteria and subsequent acute mastitis [2], [3]. Neutrophilic inflammation in the MG is driven by the detection of bacteria and bacterial components such as toxins or the so-called Microbe-Associated Molecular Patterns (MAMPs) by sensors of the innate immune system [4]. Besides this relatively non-specific innate immunity, adaptive immunity can also contribute to milk leukocytosis through antigen-specific inflammation. The recruitment of neutrophils into the lumen of the bovine mammary gland by infusion through the teat canal of a few micrograms of a protein antigen such as ovalbumin can be obtained readily by systemic immunization of dairy cows [5]. This phenomenon can be elicited with a bacterial antigen and results in the increased bactericidal efficiency of the recruited neutrophils [6]. Attempts have been made to delineate the mechanism of the antigen-specific inflammation, also called antigen-specific reaction (ASR), to proteins infused in the lumen of the MG of cows or laboratory rodents. Experiments with adoptively sensitized guinea pigs have shown that lymphocytes, but not immune serum, made recipient animals responsive to the sensitizing antigen [7], [8]. The authors concluded that milk leukocytosis in the antigen-challenged glands of sensitized animals was a local manifestation of cell-mediated immunity (CMI). The lymphocytes responsible for the adoptive transfer of CMI were not characterized, and the mechanisms of antigen-specific inflammation in the MG have not been identified so far. Nevertheless, CD4+ T cells have been shown to be required for antigen-specific recruitment of neutrophils [9]. More recently, a lineage of helper T lymphocytes which appears to be specialized in the recruitment of neutrophils at epithelial surfaces has been identified, and this newly recognized Th17 lineage is now considered a major actor of the mobilization of neutrophils and a modulator of innate and antigen-specific inflammation, both acute and chronic [10], [11]. In other settings, implication of Th17 cells has been demonstrated in the vaccine response to mucosal infections caused by *Mycobacterium tuberculosis*, *Bordetella pertussis*, and *Streptococcus pneumoniae* [12]–[14]. Upon activation, these cells produce hallmark cytokines (IL-17A, IL-17F, IL-22 and IL-26) that act on stromal cells such as fibroblasts and epithelial cells, inducing in turn the secretion of chemokines attracting and stimulating neutrophils, as well as favoring granulopoiesis [10]. In a previous study, we detected IL-17 mRNA in bovine milk leukocytes isolated from cases of mammary ASR [15]. We also found that the receptor for IL-17A and IL-17F is expressed in the bovine MG, and that mammary epithelial cells (MEC) respond to these two cytokines by producing chemokines and self-defense proteins [16]. Consequently, we hypothesized that Th17 lymphocytes and IL-17A and IL-17F could contribute to the mammary ASR induced by sensitization. One major interest of mammary ASR is that it could be used to modulate the early response of the MG to bacterial intrusion, thus improving the efficiency of MG defenses.

The purpose in the present investigation was to characterize the antigen-specific inflammation induced in MG by infusion of a sensitizing protein. Our attention was focused on particular cytokines as indicators of T-cell mediated hypersensitivity. To study mammary ASR independently of the inflammation triggered by MAMPs, we used a protein unrecognized by the innate immune system to immunize animals naïve to this antigen. Ovalbumin has proved to meet these conditions, and to be an efficient antigen to induce an antigen-specific inflammation in the bovine MG [5]. IL-17A has been shown to play a major role in the neutrophil influx into airways of ovalbumin-sensitized mice [17]. In line with the supposed contribution of Th17 lymphocytes to the mammary ASR, we tested the effect of the adjuvant effect of lipoteichoic acid and muramyl dipeptide with a view to optimizing the response by favoring Th17 elicitation, as these MAMPs have been shown in humans to orient the immune response towards this direction [18], [19]. The results obtained showed that the lymphokines IL-17A, IL-17F and IFN-γ were produced in the MG during ASR. Importantly, IL-17A was detected in milk and mammary tissue of the challenged glands at an early phase of the inflammatory response. Unexpectedly, IL-17A was found associated to MEC in control and antigen-challenged glands. These results constitute a strong incentive to identify and characterize the cells responsible for IL-17A production in the MG and to delineate the contribution of this cytokine to MG defense.

## Materials and Methods

### Experimental Scheme

The use and care of all animals in this study were approved by the Institutional Animal Care and Use Committee (CREEA, Comité Régional d’Ethique et d’Expérimentation Animale, permit number CL2007-47). Twenty-one healthy cows of our institutional experimental herd (PFIE, Nouzilly) were selected on the basis of low somatic cell counts (<200 000 cells/ml milk), absence of intramammary infection by major pathogens (*Staphylococcus aureus*, *Escherichia coli* and streptococci), and the occurrence of at least two uninfected quarters (no detectable bacterial growth). Subclinical infection by coagulase-negative staphylococci was tolerated in one or two quarters, but these infections were cleared by antibiotherapy if needed (new infections during the experimental period). Challenged and control quarters were not infected and shed less than 100 000 cells/ml milk at time of challenge. Cows, in their 1^st^ to 3^rd^ lactations and between 4 to 7 months in lactation, were distributed as evenly as possible between the different experimental groups.

In the first experiment, cows were allocated to 3 groups of 7 animals. The cows of one group received 3 subcutaneous injections of 50 µg ovalbumin in 0.5 ml phosphate buffered saline (PBS) emulsioned in 0.5 ml Freund’s incomplete adjuvant (FIA; Sigma), 5 to 6 weeks apart in the dewlap. A second group of cows were given the same immunization regime, except that the FIA was supplemented with 20 µg lipoteichoic acid (from *S. aureus*, InvivoGen, Toulouse, France) and 20 µg muramyl dipeptide (MDP, InvivoGen). The third group received the complemented adjuvant without ovalbumin. Four weeks after the second booster injection, all the cows were challenged with intramammary infusion into one quarter of 25 µg ovalbumin, and the reaction was monitored for 4 days by clinical examination and milk sampling. Just before challenge, and at each sampling time, rectal temperature was measured.

**Figure 1:**
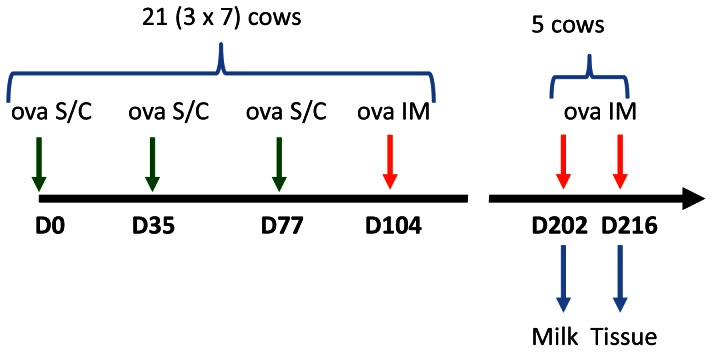
Experimental scheme. See M&M section for explanation. Green arrows indicate immunizations by subcutaneous injections of ovalbumin. Red arrows signal intramammary challenges with ovalbumin.

In the second experiment, 5 of the cows that exhibited an inflammatory response to the ovalbumin intramammary challenge were challenged again with intramammary infusion of 25 µg ovalbumin 14 weeks later (Figure 1). Milk samples were taken for 3 days in order to collect milk cells for transcriptomic analysis.

In the third experiment, the same 5 cows were challenged again in another quarter 2 weeks later with 25 µg ovalbumin in order to collect mammary tissue. Tissue samples were collected about 14 h after ovalbumin infusion in the infused quarter and in an uninfused (control) healthy quarter which was neither infected nor previously challenged with ovalbumin. Just after slaughter with a penetrating captive bolt gun followed by exsanguination, mammary tissue was taken in the secretory region just above the gland cistern. For each quarter, a piece of tissue (ca. 2 cm^3^) was removed and fixed in neutral-buffered 4% formalin for immunohistochemistry (IHC). Smaller pieces were taken, dipped in 800 µl of RNA Later solution (Ambion), wrapped in aluminum foil and snap frozen in liquid nitrogen and stored at −80°C for RNA extraction.

### Challenge with Ovalbumin

Cows were challenged by infusing ovalbumin into the lumen of one quarter through the teat canal. Pyrogen-free ovalbumin (Calbiochem) was made soluble at an approximate concentration of 2 mg/ml in Hank’s buffered salt solution (HBSS), this solution was sterile-filtered (0.2 µm) and adjusted to the final concentration of 25 µg/ml on the basis of the optical density taken at 280 nm (absorbance of 1 mg/ml = 0.74) by dilution in HBSS supplemented with 1 mg/ml bovine serum albumin (BSA, cell culture tested, Sigma). BSA was used as a vehicle to prevent the adsorption of ovalbumin onto the pyrogen-free vessel walls. This procedure was repeated before each challenge experiment owing to the propensity of ovalbumin to aggregate when in solution. Immediately following the morning milking, 1 ml of the freshly made ovalbumin solution (25 µg) was infused through the teat canal by means of a sterile smooth cannula fitted to a 1-ml syringe whose plunger was vigorously pushed. Then the teat was massaged upward to ensure the diffusion of the ovalbumin dose.

### Examination of Milk Samples

Aseptically taken foremilk samples were collected prior to challenge and 8, 12, 24, 32, 48, 72 and 96 h post-infusion (hpi) with ovalbumin. Bacterial analysis was performed throughout the experiments as previously described by plating 20 µl milk over sheep blood-esculin agar, and milk cell counts were measured with an automated cell counter (Fossomatic model 90; Foss Food Technology, Hillerod, Denmark) as described [20]. Proportions of neutrophils and mononuclear cells were determined by microscopic examination of cytospin slides prepared and stained with May-Grünwald-Giemsa as described [6]. At least 200 cells per smear were examined and categorized as neutrophils or mononuclear cells. Then the milk samples were centrifuged at 1,500×*g* for 30 min at 4°C. Skimmed milk between the supernatant (fat layer) and the pellet (debris and cells) was harvested and stored in portions at −18°C. In experiment 2, RNA was extracted from cell pellets. Milk samples were diluted 1/5 in PBS and centrifuged at 1,500×*g* for 10 min at 4°C. After microscopic evaluation of cell numbers, about 10^7^ cells were lysed with 750 µl of lysing buffer (NucleoSpin RNA II extraction kit; Macherey-Nagel, Düren, Germany), immediately snap-frozen in liquid nitrogen and stored at −70°C.

### ELISAs for C5a, CXCL3, CXCL8, TNF-α, IFN-γ, IL-1β, and IL-6

Enzyme-linked immunosorbent assays (ELISAs) for the complement fragment C5a, the chemokines CXCL3 (GRO gamma), CXCL8 (IL-8) and TNF-α were performed as previously described [21]–[24]. Milk IFN-γ concentrations were determined with a commercially available bovine IFN-γ ELISA kit (MAbtech AB, Nacka Strand, Sweden). To determine concentrations of IL-1β and IL-6, commercially available kits for bovine cytokines were used according to manufacturer’s instructions (Thermo Scientific, Rockord, IL, USA). The lower limits of detection for IL-1β, IL-6, IFN-γ and TNF-α in milk by the ELISAs were 60 pg/ml, 350 pg/ml, 10 pg/ml and 40 pg/ml, respectively.

### ELISA for Antibodies to Ovalbumin

Total antibodies to ovalbumin were measured in serum by indirect ELISA. Microtiter plates (Nunc Immunoplate Maxisorp) were coated by overnight incubation with 5 µg/ml ovalbumin (100 µl/well) in phosphate-buffered saline (PBS), before saturation with a solution of 5 mg/ml fish gelatin in PBS (PBSG) for 1 h at 37°C. Sera were diluted 1/100 to 1/20000 in PBSG and incubated for 2 h at 37°C. Following each incubation step, the plates were washed three times with PMS supplemented with 0.1% (v/v) Tween 20 (Sigma). After incubation for 30 min at 37°C with a 1/20000 dilution of goat anti-bovine IgG (H+L) conjugated to horseradish peroxidase (Jackson ImmunoResearch Laboratories), the reaction was revealed with 3,3′,5,5′-tetramethylbenzidine enzyme substrate (Uptima, Interchim, Montluçon, France). To determine antibody concentrations, a standard curve was established in each plate with a series of dilutions of bovine serum antibodies to ovalbumin affinity-purified with the antigen. Concentrations of antibodies were calculated with the software provided with the ELISA reader manufacturer (Labsystems, Helsinki, Finland).

### Antibodies to Bovine IL-17A

Polyclonal antibodies to bovine IL-17A were generated by immunizing rabbits with synthetic peptides (synthetized by PolyPeptide group, Strasbourg, France) corresponding to the N-terminus (GVIIPQSPG) or the C-terminus (VTPIVRHLA) sequences of IL-17A coupled to hen ovalbumin following a protocol previously described [23]. Antibodies (designated C-term and N-term) were affinity-purified using the peptides coupled to EAH-Sepharose 4B (GE-Healthcare) and some N-term antibodies were biotinylated as described [23]. Rabbit antibodies to hen ovalbumin were affinity-purified using ovalbumin coupled to EAH-Sepharose. Commercial affinity-purified antibodies to whole recombinant bovine IL-17A were from Kingfisher Biotech (St Paul, MN, USA), and rabbit antibody (ab79056) to human IL-17A, raised against a synthetic 19 amino acid-peptide from near the center of human IL-17A and affinity-purified with the immunogen, was from Abcam. The reactivity of IL-17A N-term, C-term and whole protein antibodies was checked by western blotting after SDS-PAGE using recombinant bovine IL-17A and IL-17F prepared as described [15], [16]. All four antibodies reacted with IL-17A (one band in the 17 kDa region), but not with IL-17F (results not shown).

### ELISA for IL-17A

Reactions (100 µl) were performed in flat-bottom 96-well plates (Nunc Immunoplate Maxisorp). Following each incubation step, the plates were washed three times with PBS supplemented with 0.1% (v/v) Tween 20 (Sigma) with an automatic washer (Beckman Coulter, Fullerton, CA, USA). Dilution of sera and reagents were done in PBS containing 0.1% gelatin (PBSG) (Gibco). The sequence of incubation steps was as follows: (1) rabbit antibodies to bovine IL-17A (Kingfisher Biotech) (1 µg/ml in PBS 0.1 M pH 7.3) overnight at 4°C; (2) blocking with 0.5% gelatin in PBS for 30 min at 37°C; (3) samples under test (used neat or half diluted in PBSG) and a series of dilutions of insect cell culture supernatant containing a known concentration of recombinant bovine IL-17A to establish the standard curve, for 2 h at 37°C; the concentration of the IL-17A in the insect cell culture supernatant obtained as described [15] was determined with reference to commercialized recombinant bovine IL-17A (Kingfisher) used as standard in the ELISA, and was preferred to this latter owing to its better stability. (4) incubation with 1 µg/ml biotinylated affinity-purifed Ab to the N-terminal peptide of bovine IL-17A for 1 h at 37°C; (5) incubation with peroxidase-conjugated avidin (Neutravidin, Molecular Probes) diluted 1/20 000 for 30 min; (6) incubation with 3,3′,5,5′-tetramethylbenzidine (TMB) for ELISA (Uptima, Interchim, Montluçon, France). Optical densities at 450 nm minus the absorbance at 540 nm were measured with a Multiskan RC reader (Labsystems, Helsinki, Finland) after incubation at room temperature and addition of stop solution (1.0 M HCl). Concentrations were calculated with the Genesis software (Labsystems). The intra-assay variability was determined by testing replicates of a few samples and of standard dilutions of recombinant IL-17A. The intra-plate and inter-plate coefficients of variations were less than 15%. This ELISA did not cross-react with bovine IL-17F as checked by using recombinant bovine IL-17F [16] as antigen.

### Reverse Transcription and qPCR Analysis

Before RNA extraction, 100 mg of mammary tissue (first minced with a scalpel) were homogenized in a tube with ceramic beads 2.8 mm in diameter (Mobio) and 700 µl of lysing buffer with a cell disrupter (Fast Prep FP120– ThermoSavant). Total RNA was extracted from milk cells or mammary tissue by using the NucleoSpin RNA II extraction kit (Macherey-Nagel, Düren, Germany), and the residual genomic DNA was removed by using DNase digestion with RNase-free DNase (Macherey-Nagel). The total RNA quantity was assessed by using a NanoDrop spectrophotometer (NanoDrop Technologies, Wilmington, DE). RNA integrity was analyzed using the Agilent Bioanalyzer System (Agilent technologies, Inc., Santa Clara, CA, USA). RNA samples yielding a RIN (RNA Integrity Number) <6.5 were discarded. Total RNA (1 µg) was then reverse transcribed to cDNA using random hexamers and SuperScript RT III (Invitrogen) according to manufacturer’s instructions. Diluted cDNA samples were stored at 4°C until use. Primers used in this study are listed in Table 1. Relative quantities of gene transcripts were measured as described previously [25] with a Bio-Rad Chromo 4 detection system (Bio-Rad, Hercules, CA, U.S.A.). Briefly, qPCR data were normalized against 3 reference genes (*Actb*, *PPIA*, *18S rARN*) according to [26], and the expression of each gene was worked out relative to the sample giving the lowest value which was arbitrarily set to 1. Of note, this precludes the comparison of relative expression between genes.

**Table 1:** Gene-specific oligonucleotide primers used for qPCR.

Gene Symbol	Oligonucleotides (5′–3′) F:forward; R: reverse	Amplicon(bp)	AnnealingTemperature(°C)	Accession number(GenBank)
*18s rRNA*	F: CGGGGAGGTAGTGACGAAA	196	69	AF176811
	R: CCGCTCCCAAGATCCAACTA			
*Actb*	F: ACGGGCAGGTCATCACCATC	166	67	BT030480
	R: AGCACCGTGTTGGCGTAGAG			
*PPIA*	F: TCCGGGATTTATGTGCCAGGG	206	67	BC105173
	R: GCTTGCCATCCAACCACTCAG			
*Il17a*	F: GCCCACCTACTGAGGACAAG	246	62	NM_001008412
	R: GCTGGATGGTGACAGAGTTC			
*Il17f*	F: CACTCTGGAGGACCACATTG	216	62	XM_582420
	R: GAGTTCAGGGTCCTGTCTTC			
*Il21*	F: GTGGCCCATAAGTCAAGCTC	152	62	NM_198832
	R: CGCTCACAGTGTCTCTTTAC			
*Il22*	F: AGGAGCCCTACATCTTCAAC	122	62	NM_001098379
	R: CTTCGTCACCTGATGGATTC			
*Il26*	F: CAGAGCAACGATTCCAGAAG	194	62	XM_001250651
	R: TCTGCCTGAGGCTATGAAAG			
*Ifng*	F: TGCAAGTAGCCCAGATGTAG	213	62	NM_174086
	R: CAGAGCTGCCATTCAAGAAC			
*Il12a*	F: ACAGAAGGCCAGACAAACTC	150	62	NM_174355
	R: AGC CAG ACA ATG CCC ATT AG			
*Il12b*	F: CACCAGCAGCTTCTTCATCA	232	62	NM_174356
	R: CTTGTGGCATGTGACTTTGG			
*Il23a*	F: GATGGCTGTGATCCACAAGG	235	62	XM_588269
	R: TGGGAATAGGGCTTGGAGTC			
*Ccl2*	F: GCTCGCTCAGCCAGATGCAA	117	62	NM174006
	R: GGACACTTGCTGCTGGTGACTC			
*Ccl5*	F: CTGCCTTCGCTGTCCTCCTGATG	217	62	NM175827
	R: TTCTCTGGGTTGGCGCACACCTG			
*Ccl20*	F: TTCGACTGCTGTCTCCGATA	172	62	NM174263
	R: GCACAACTTGTTTCACCCACT			
*Cxcl10*	F: TTCAGGCAGTCTGAGCCTAC	218	62	NM_001046551
	R : ACGTGGGCAGGATTGACTTG			
*Nos2*	F: CTTGAGCGAGTGGTGGATGG	240	64	NM001076799
	R: CCTTCATCCTGGACGTGGTTC			
*Saa3*	F: CCTCAAGGAAGCTGGTCAAG	226	62	NM181016
	R: TACCTGGTCCCTGGTCATAC			
*TAP*	F: GTAGGAAATCCTGTAAGCTGTG	139	64	AF014106
	R: GTGTCTTGGCCTTCTTTTAC			

### Immunohistochemistry for the Detection of IL-17A in Mammary Tissue

Tissue samples were fixed in 4% formalin for at least 1 week before paraffin-embedding. Tissue sections (5 µm thick) were cut and collected onto treated glass slides (Superfrost Plus, Menzel-Glaser) and dried for 2 days at 37°C, then overnight at 56°C, before being deparaffinized and rehydrated. An antigen retrieval step was performed by autoclaving tissue sections in 10 mM pH 6.1 citrate buffer for 15 or 30 min at 121°C. Endogenous peroxidase was inhibited using a 1/100 dilution of 30% hydrogen peroxide (Sigma) in methyl alcohol for 30 min at room temperature. Washing steps were performed with tap water. Non-specific binding sites were blocked with a 20 min incubation in 50% normal goat serum and 50% fetal calf serum (Gibco, InVitrogen corporation). Working concentrations of the first antibodies were determined in preliminary experiments by assaying increasing dilutions of antibodies. C-term, N-Term and ab79056 antibodies were used at concentrations of 5, 1.25 and 1.25 µg/ml, respectively. Immunolabeling of mammary tissue sections by incubation with these primary antibodies for 60 min was followed by a 30 min incubation with the universal immuno-peroxidase polymer, anti-mouse and -rabbit N-Histofine® (Nichirei Biosciences Inc, Microm Microtech France). Enzymatic activity was revealed using diaminobenzidin (DAB+, LabVision). Each step was followed by washes with 1% BSA (Uptima) and 0.05% Tween 20 in PBS. Sections were counterstained with a Harris’ hematoxylin solution. Secondary-antibody control was routinely performed with the antibody-diluting buffer used in place of the primary antibody. Isotypic antibody control was performed with rabbit antibodies to ovalbumin (at 4.25 µg/ml) affinity-purified on the antigen fixed on a AH-Sepharose gel using the same protocol as for the antibodies to IL-17A peptides.

To check the specificity of the labeling by the N-term antibody, it was pre-incubated at its working concentration with its antigen peptide at 66 µM for 1 h at room temperature before incubation with mammary tissue sections.

### Statistical Analysis

Most of the statistical analyses were performed with the StatXact software (Cytel software Corp., Cambridge, MA, USA), using non-parametric tests. A probability level of <0.05 was considered statistically significant. Milk cell concentrations between groups were compared with the permutation test for independent samples (two-sided). The significance of concentration variations of cytokines and chemokines with time were tested with the Friedman test, and the significance of differences of antibody concentrations with the Mann & Whitney test. The significance of the difference of cytokine expression in mammary tissue of challenged and control quarters was tested with the permutation test for paired samples. Correlations between cell concentrations and milk concentrations of chemoattractants and cytokines were calculated with the Bravais-Pearson test (Excel software).

## Results

### Antigen-induced Clinical Signs (Data Not Shown)

Cows were split between 3 groups of 7 animals which received 3 subcutaneous injections of ovalbumin with two different adjuvants or received adjuvant without antigen (Figure 1). Following intramammary infusion of ovalbumin into one quarter of the immunized cows, local and systemic signs of inflammation developed in 9 of the 14 immunized cows but not in any of the mock-immunized animals. Elevated rectal temperatures were detected in the responsive cows at 12 hpi, peaked at 24 hpi (39.3 to 41.0°C) and returned to normal by 48 hpi. Local signs of inflammation in the infused quarter of responsive cows were visible at 24 hpi up to 72 hpi with some redness, a moderate swelling and some tenderness. Signs of mastitis had disappeared at 96 hpi. Milk alterations (small clots) became obvious in these quarters at 24 hpi, but greatest changes (flakes) were usually seen at 48–72 hpi. Milk appearance was back to normal by 96 hpi. Quarters adjacent to reactive quarters were visually normal. During the experimentation, none of the cows contracted an infection in the challenged or control quarters.

### Influx of Leukocytes into the Lumen of the Mammary Gland

**Figure 2:**
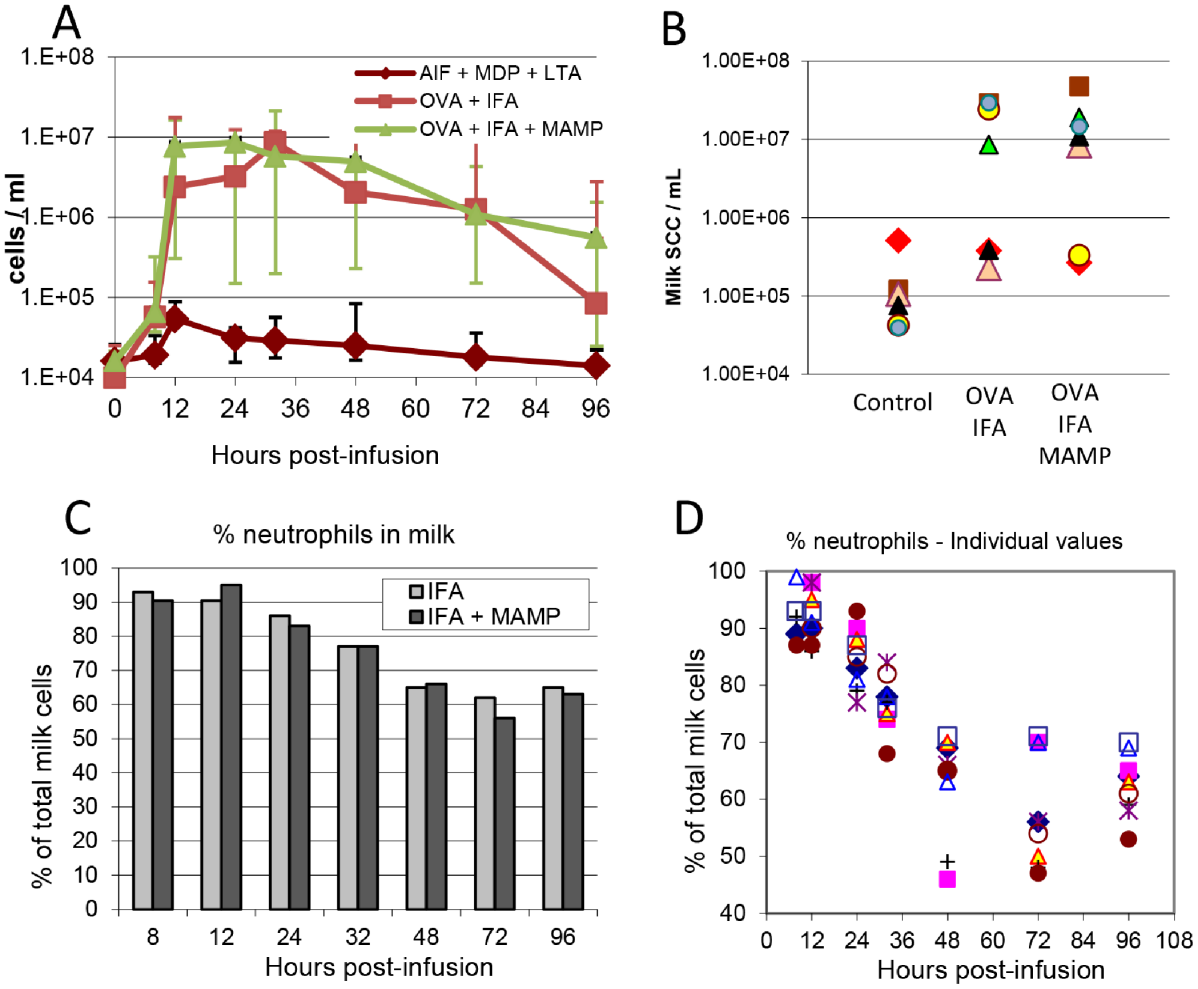
Influx of leukocytes following the intramammary challenge of 21 cows with ovalbumin. A) Kinetics of cell concentrations in milk of unimmunized control cows, cows immunized with ovalbumin in incomplete Freund adjuvant, or cows immunized with ovalbumin in IFA supplemented with MAMP (MDP and LTA) (n = 7 per group); B) Individual values of milk cell concentrations at the peak of the cellular recruitment (3×7 cows); C) percentage of neutrophils (median values), determined by examination of cytospin slides stained with May-Grünwald-Giemsa, of the cows that reacted following immunization with ovalbumin in IFA (IFA; n = 4) or immunization with ovalbumin in IFA supplemented with MAMP (IFA+MAMP; n = 5); D) Kinetics of the individual values of the 9 immunized cows that reacted to ovalbumin challenge without distinction of groups.

Intramammary infusion of 25 µg ovalbumin into one quarter of the cows previously immunized with this antigen induced a marked recruitment of cells in milk, whereas cellular recruitment was comparatively transient and anecdotal in the quarters of unimmunized cows (Figure 2A). Milk cell concentrations (MCC) increased by 8 hpi and peaked between 12 and 36 hpi. Overall, MCC reached a plateau between 12 and 48 hpi and then gradually decreased. The kinetics and magnitude of cell recruitment were comparable between cows immunized with ovalbumin whatever the adjuvant, and the differences were not statistically significant (p = 0.94, permutation test). A salient feature was the variability of the response between cows: five immunized cows did not respond more than the controls, whereas the other 9 immunized animals reacted with a peak response ranging from 8.5 to 47.6 million cells/ml milk (Figure 2B). The picture was of two clear-cut groups among the 14 immunized cows, one group of 9 responders and one group of 5 low-responders, with no apparent difference as a function of the adjuvant (Figure 2B).

**Figure 3:**
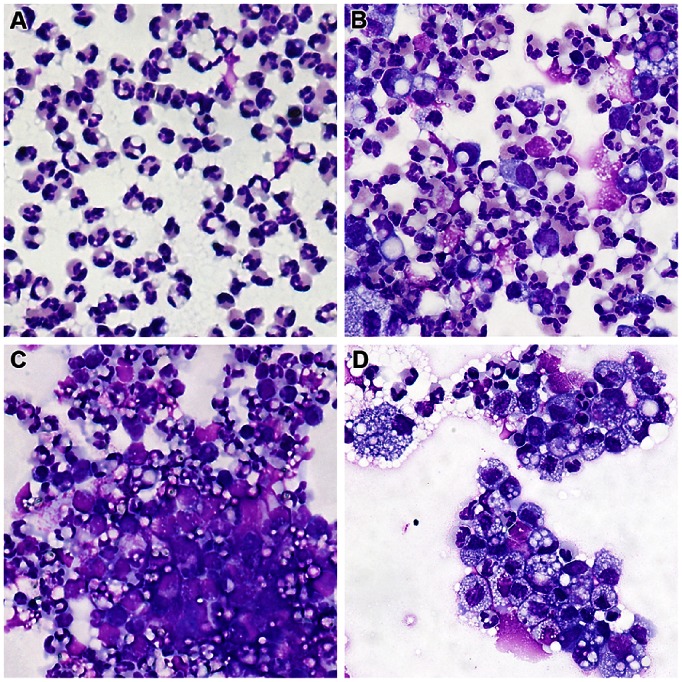
Microscopic examination of milk cells recruited at different times following intramammary infusion with ovalbumin. Milk cells from quarters infused with ovalbumin were cytocentrifuged on glass slides and stained with May-Grünwald-Giemsa. Slides representative of each sampling time are shown. A) 12 hpi; B) 24 hpi; C) 48 hpi; D) 96 hpi;

Percentages of neutrophils among recruited cells were assessed by centrifuging milk samples on glass slides (cytospins) and staining with May-Grünwald-Giemsa. Microscopic examination revealed that on average about 90% of recruited cells were neutrophils at the onset of inflammation (8–12 hpi), and that this proportion decreased by 24 hpi to reach about 60% by 72 hpi (Figure 2C). There was no obvious and statistically significant (p = 0.49, Permutation test) difference among responder cows as a function of the adjuvant. It was difficult to establish the proportions of lymphocytes, monocytes and macrophages because most cells were present in aggregates (Figure 3). It appeared that epithelial cells were only seldom seen in the smears at any time post-challenge.

**Figure 4:**
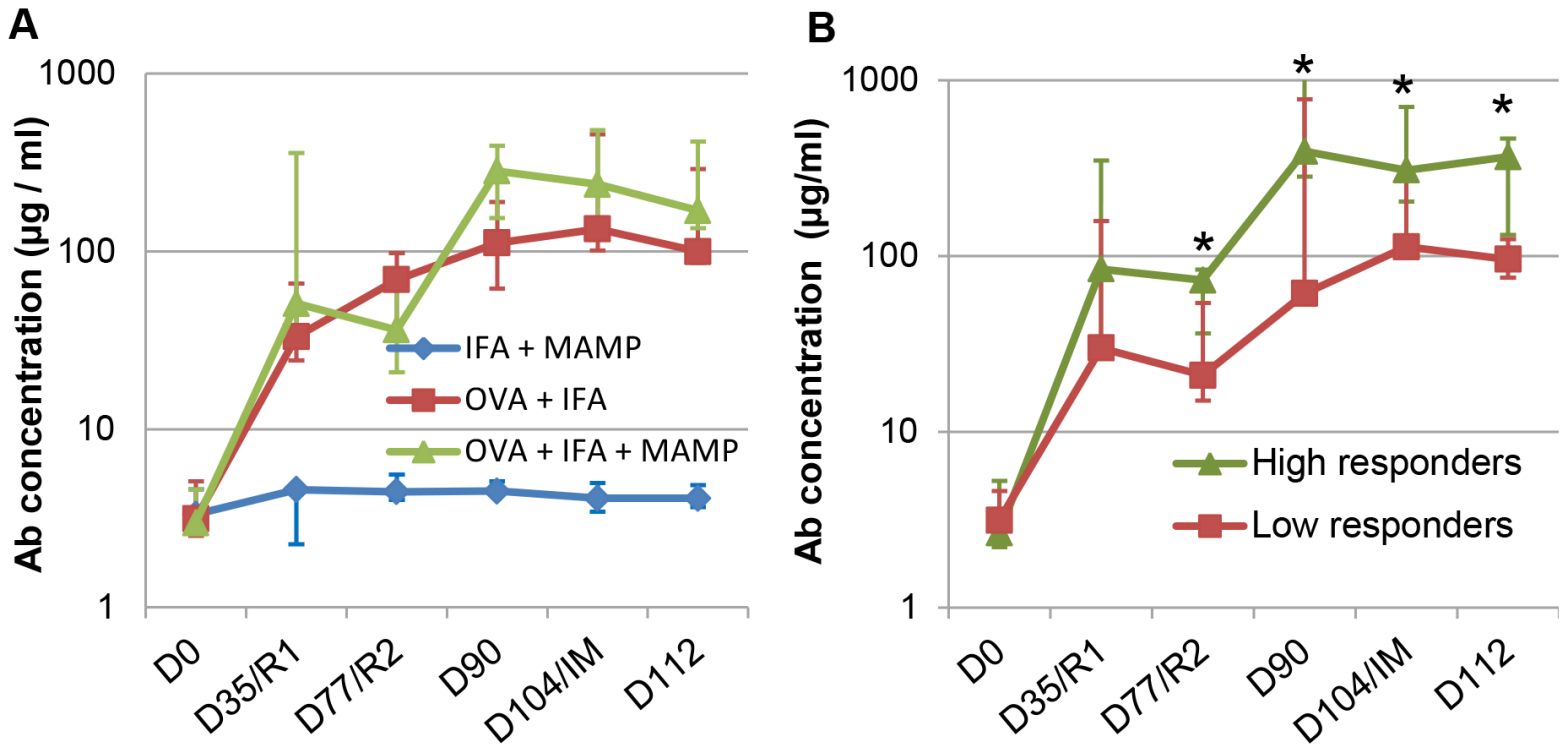
Concentrations of antibodies to ovalbumin following immunization. Data are presented as a function of the immunization group (A) or of the mammary ASR category following intramammary ovalbumin challenge (B). Antibodies were quantified in serum by ELISA before immunization (D0), on the day of first recall (D35/R1), on the day of second recall (D77/R2), two weeks later (D90), just before the intramammary challenge with ovalbumin (D104/IM) and eight days later (D112). Antibody concentrations did not differ significantly between the OVA+IFA and OVA+IFA+MAMP groups. Concentrations differed significantly between the high and low responder groups when indicated (*; Mann & Whitney test).

The quantification of the serum antibodies to ovalbumin revealed that all of the immunized cows had developed antibodies, whether high or low responders to the intramammary challenge. Mock-immunized cows did not develop antibodies, and the difference between the two groups immunized with ovalbumin was not significant (Figure 4A). Differences in antibody titers were significant (P<0.05, Mann & Whitney test) between the group of 9 responders and the group of 5 low-responder cows (Figure 4B).

Owing to the absence of difference between the kinetics, magnitude, and proportion of neutrophils in inflammatory milk, the 9 responder cows were considered as belonging to one group, irrespective of the adjuvant used with ovalbumin for immunization, and their associated data were considered as coming from one group of 9 responders thereafter. The two groups OVA/IFA and OVA/IFA+MAMP were merged on the assumption that the supplementation of IFA with MDP and LTA had not noticeably modified the cell-mediated immune response of the recipient animals. Considering the evolution of the proportion of cells in the milk of the 9 cows sensitized to ovalbumin, it appeared that individual variations were obvious as soon as 8 hpi, with animals showing up to 14% of mononuclear cells (deduced as complement to neutrophil percentages), and that the dispersion of data increased with time (Figure 2D). By 48 hpi, concentrations of neutrophils were between 45 to 70% and remained at this level up to the end of the observation period (96 hpi).

The predominant population of neutrophils displayed lobulated nuclei and most cells were dispersed individually up to 12 hpi (Figure 3A). Thereafter, cells tended to aggregate and clusters of neutrophils and mononuclear cells comprised the majority of cells (Figure3 B–F). The appearance of mononuclear cells was very diverse in size, nucleus/cytoplasm ratio and cytoplasmic staining. Although this cannot be ascertained with a simple MGG staining, cells with the appearance of lymphocytes, monocytes and macrophages were visible in high numbers. The conspicuous absence of eosinophils throughout the study in every sample is noteworthy, all the more so as most of the cows had noticeable eosinophilia, with blood eosinophil concentrations ranging from 10 to 35% of the granulocyte fraction (result not shown).

### Chemoattractants and Pro-inflammatory Cytokines in Milk of responder cows

**Figure 5:**
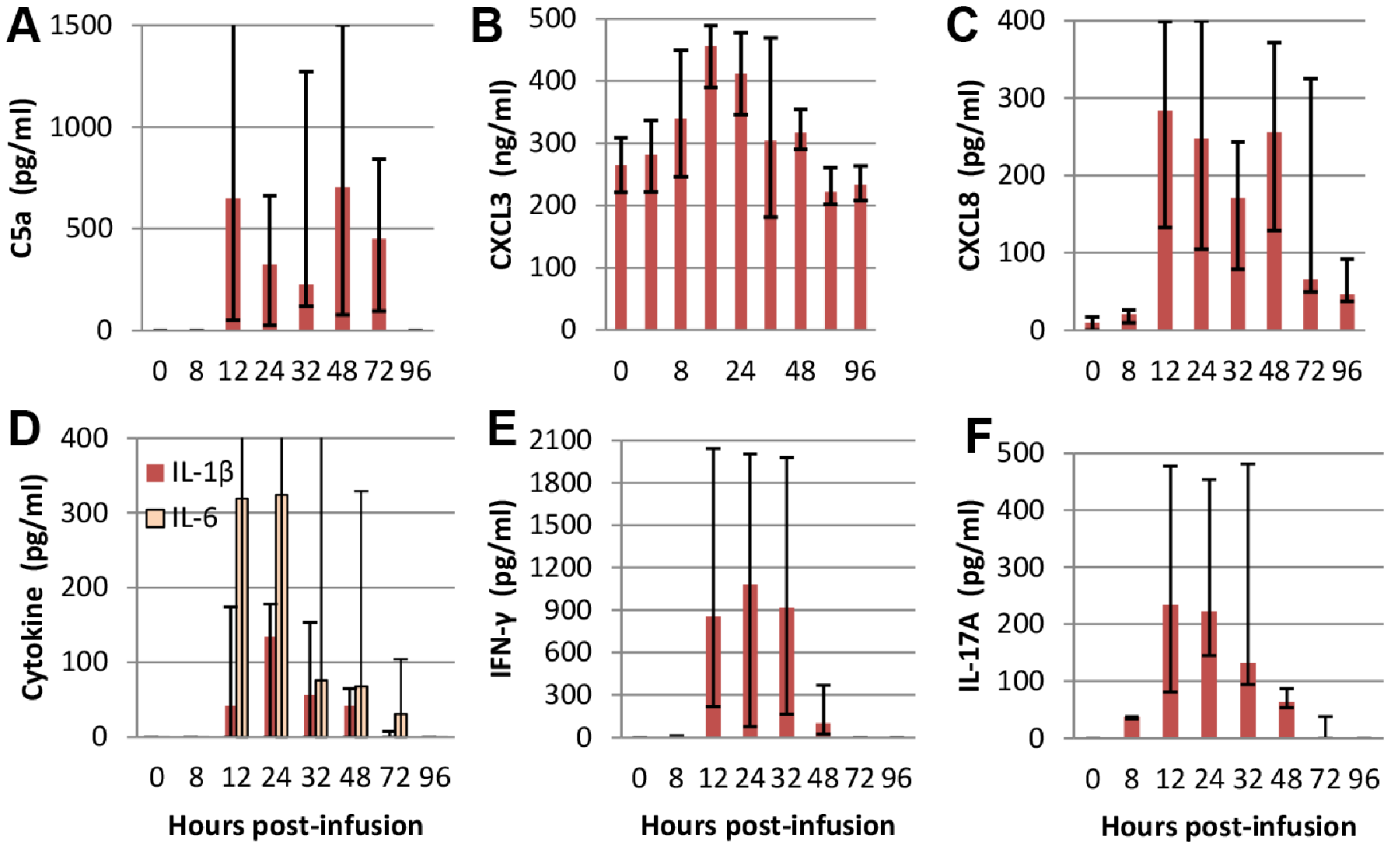
Concentrations of chemoattractants and cytokines in milk samples of the 9 responsive cows. Concentrations were measured by ELISA in the milk samples of the 9 responder cows. Quarters were infused with 25 µg ovalbumin at time 0 and milk samples taken at indicated times. Median values (Q1, Q3) are shown. Concentrations varied significantly (Friedman test) as a function of hpi for C5a, CXCL8, IL-1β, IL-6, IFN-γ, IL-17A (p<0.001) and CXCL3 (p = 0.002).

The complement-derived chemoattractant C5a was detected in the milk of the 9 responder cows, but with highly variable concentrations, and not until 12 hpi (Figure 5A). Concentrations of the chemokine CXCL3 were already high (265 ng/ml) at the time of infusion, as expected since it is constitutive in bovine milk [23], but it increased to 456 ng/ml at its peak at 12 hpi (Figure 5B). Concentrations of CXCL8, which were hardly detectable at 0 hpi, increased slightly at 8 hpi and augmented abruptly at 12 hpi (Figure 5C). Nevertheless, CXCL8 concentrations remained extremely low with median values less than 300 pg/ml, more than a thousand fold less than CXCL3 concentrations. The cytokines IL-1β and IL-6 were not detected until 12 hpi (Figure 5D). Concentrations remained low and conspicuously variable between animals, with undetected levels in 3 and 4 cows for IL-1β and IL-6, respectively. The cytokine IFN-γ was detected in the milk of the 9 responder cows, but with ample concentration variations between animals, and not until 12 hpi for 5 cows (Figure 5E). Concentrations remained noticeable until 32 hpi, and then decreased abruptly. Interestingly, the cytokine IL-17A was also detected in the milk of all the responder cows as soon as 8 hpi. Thereafter, concentrations suddenly increased at 12 hpi and then began to decrease sharply to disappear by 72 hpi in most animals (Figure 5F). TNF-α was not detected in any of the milk samples. Except for CXCL3, none of the investigated chemokines and cytokines was found in milk samples from control quarters of mock-immunized cows.

The correlations between the chemoattractants and the two pro-inflammatory cytokines whose concentrations increased in all the 9 cows were calculated (Bravais-Pearson test) over two time periods, the entire follow-up period, and the early phase of the inflammatory response (0–24 hpi). Among the chemoattractants, the best correlation between variations of concentrations and MCC was for CXCL3 at the onset of inflammation (Table 2). C5a concentrations were loosely correlated with MCC variations, and CXCL8 concentrations correlated poorly even at the beginning of the cellular response. The best correlations were observed with INF-γ and IL-17A, both at the beginning and throughout the observation period (Table 2).

**Table 2:** Correlations between milk cell concentrations (MCC) and concentrations of the chemoattractants and pro-inflammatory cytokines that were detected in the milk of all the responder cows.

	Chemoattractants or chemokines				
	C5a	CXCL3	CXCL8	IFN-γ	IL-17A
MCC 0–24 hpi	0.466	0.708	0.131	0.878	0.750
MCC 0–96 hpi	0.262	0.319	0.194	0.744	0.634

MCC 0–24 hpi: milk cell concentrations during the onset of the inflammatory response (0, 8, 12 and 24 h post-infusion). MCC 0–92 hpi: milk cell concentrations during the whole monitoring period. Correlations were calculated with the Bravais-Pearson test over two periods of time: at the onset of inflammation (0–24 hpi) and throughout the observation period (0–96 hpi).

### Characterization of Cytokine Expression in Milk Cells

**Figure 6:**
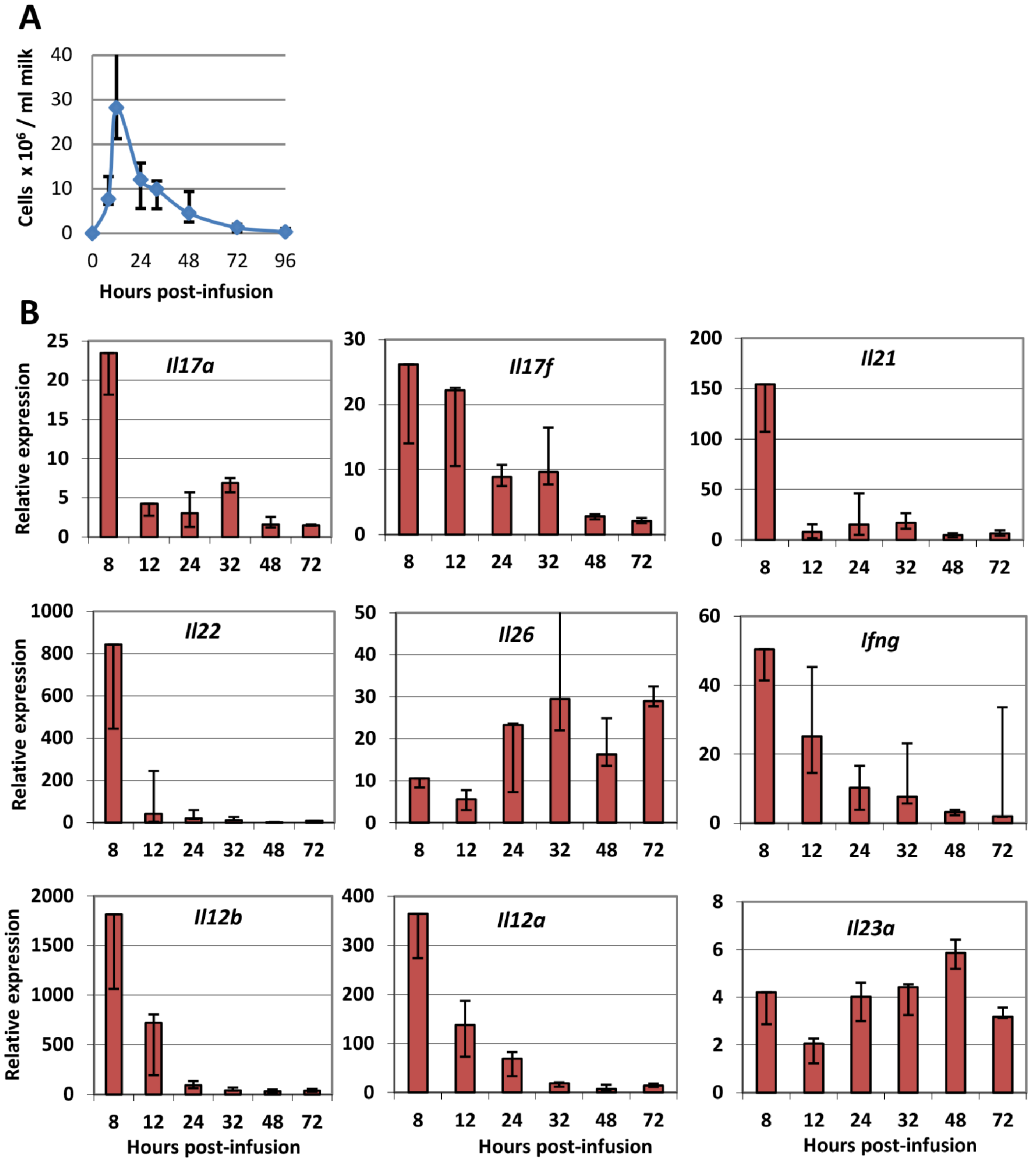
Characterization of cytokine expression in milk cells following intramammary challenge with ovalbumin. Five responder cows were challenged in one quarter with 25 µg ovalbumin and milk cells were collected for RT-qPCR. A) Kinetics of cellular influx in milk of challenged quarters. B) Kinetics of the relative mRNA expression of the indicated cytokines or cytokine components. Median values (Q1, Q3) are shown. It is important to keep in mind that relative expressions, contrary to absolute expression, do not allow comparisons between genes.

Five of the 9 responder cows were available to be restimulated by intramammary infusion of ovalbumin 14 weeks after the first challenge. All of the cows reacted by recruiting high concentrations of cells in milk with a kinetics similar to that of the previous challenge, beginning by 8 hpi and peaking at 12 hpi (Figure 6A).

Milk cells were collected and RNA extracted for analysis by RT-qPCR, and the expression of genes associated with Th17 and Th1 lymphocytes was determined (Figure 6B). All the tested cytokines were expressed in all samples, but with ample variation as a function of time post-infusion. Expression kinetics of genes encoding IL-17A, IL-17F, IL-21, IL-22 and IFN-γ were comparable: the highest expression was at 8 hpi, and then expression declined to very low levels at 72 hpi. There was some difference in that the expression of *Il21* and *Il22* plummeted whereas the expression of *Il17a*, *Il17f* and *Ifng* declined gradually (Figure 6B). The expression kinetics of *Il26* was completely different with a belated increase followed by a steady expression up to 72 hpi. The expression of some genes that have been implicated in the induction of the Th17 and Th1 signature cytokines was also monitored. The expression of *Il12b* (encoding IL-12/23p40) and *Il12a* (encoding IL-12p35), which together code for the IL-12 cytokine, was much higher at the beginning of the inflammatory response than at 24 hpi and later (Figure 6B). The expression kinetics of *Il23a* (encoding the IL-23p35 component of IL-23) was different with a rather steady expression throughout the monitored period. Due to low concentrations and viability of cells in uninfused control quarters leading to poor quality RNA, RT-qPCR could not be carried out on cells from uninflamed quarters.

### Characterization of Cytokine and Chemokine Expression in Mammary Tissue

**Figure 7:**
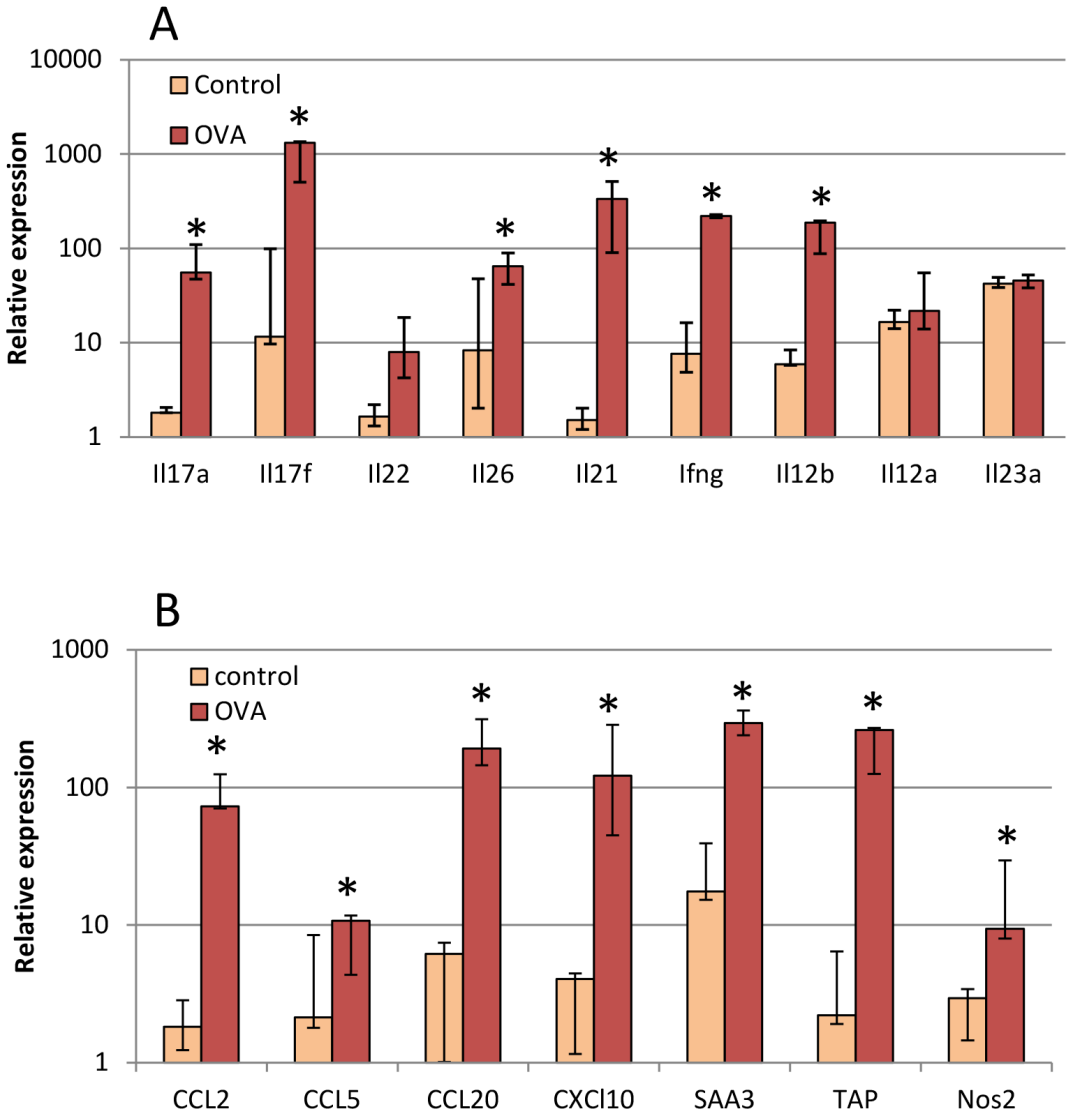
Characterization of cytokine expression in mammary tissue following intramammary challenge with ovalbumin. Five responder cows were challenged in one quarter with 25 µg ovalbumin and mammary tissue was collected for RT-qPCR about 14 h later. A) Relative expression of cytokines associated with lymphocytes or considered as stimulating the expression of these cytokines by lymphocytes. B) Relative expression of genes coding chemokines and defense components. * significant difference (p<0.05; permutation test for paired samples) between challenged and control quarters.

The 5 cows that had been restimulated by intramammary infusion of ovalbumin 14 weeks after the first challenge were challenged again by intramammary infusion of ovalbumin into one quarter two weeks after the second challenge. RNA was extracted from the mammary tissue samples taken from challenged quarters about 14 h after infusion with ovalbumin. In parallel, tissue was taken from an adjacent quarter that was never infused for comparison. Sample analysis by RT-qPCR showed a clear overexpression of genes encoding cytokines considered as characteristic of Th17 lymphocytes, i.e. *Il17a*, *Il17f*, *Il22*, *Il26* and *Il21* (Figure 7A). The expression of *Ifng* was also augmented. As it has been established that in human and mouse IL-12 induces the expression of *Ifng* by Th1 cells and IL-23 the expression of the other measured genes by Th17 cells, we investigated the expression of the components of these two cytokines in the same tissue samples. The expression of *Il12b* coding the shared component was augmented, but expressions of *Il12a* and *Il23a* coding the cytokine specific components were not (Figure 7A).

Since a sizeable proportion of cells recruited in milk was mononuclear leukocytes, the relative expression of some of the chemokines that are able to attract monocytes and lymphocytes was also determined (Figure 7B). The genes coding for CCL2, CCL5 (RANTES, regulated upon activation, normal T-cell expressed and secreted), CCL20 and CXCL10 (IP-10, interferon γ-induced protein 10 kDa ) were overexpressed, in agreement with the mononuclear cell influx. We also assessed the expression of a few genes that are upregulated in MEC in response to IL-17A or IL-17F [16]. The expression of the acute phase protein SAA3 (serum amyloid A3), the defensin TAP (tracheal antimicrobial peptide) and the enzyme Nos2 (inducible NO synthase) were all augmented in the challenged quarters compared to unchallenged controls (Figure 7B).

### Immunohistochemistry of Mammary Tissue for IL-17A Detection

**Figure 8:**
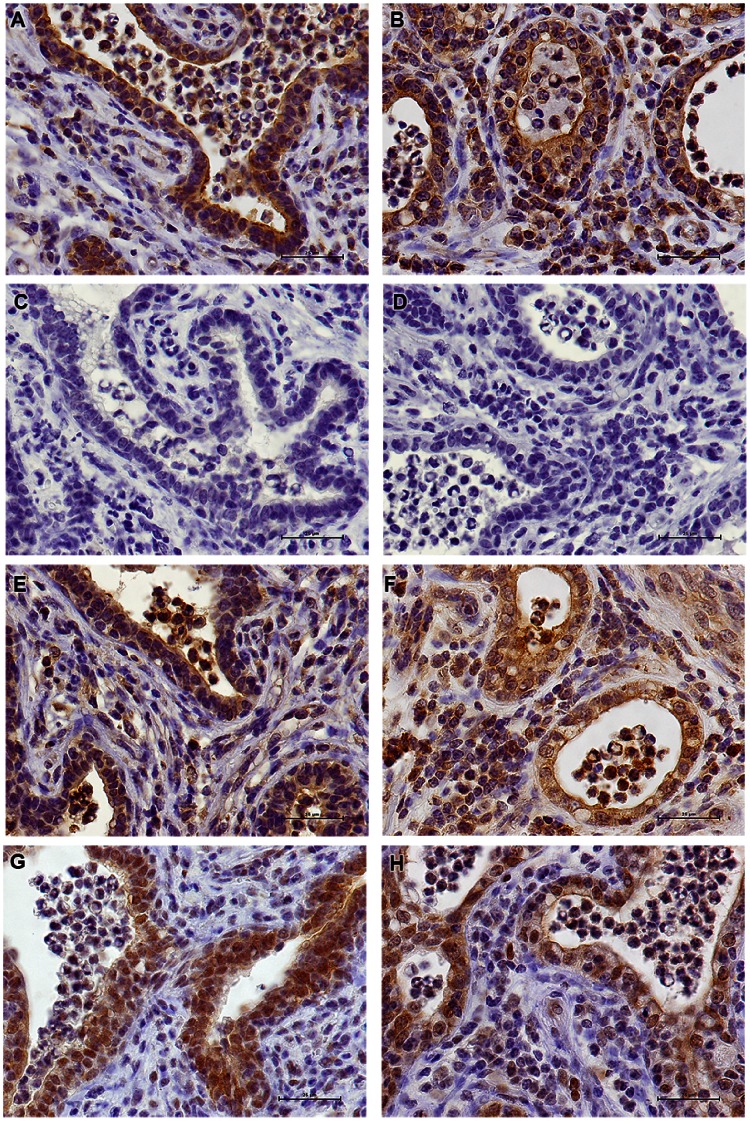
Analysis by immunohistochemistry of representative sections of mammary tissue of ovalbumin-infused glands. A) Immunoreactivity of the epithelial lining the alveoli and of cells in the connective tissue (cow #4019) to antibody against N-terminal peptide of bovine IL-17A; B) Immunoreactivity of the mammary tissue of another cow (#1039) to the N-term antibody; C) Inhibition of labeling by Ab to N-terminal IL-17A peptide with the peptide antigen (cow #4019); D) Negative control with Ab to ovalbumin and second antibody conjugated to horseradish peroxidase; E, F) Immunoreactivity of the epithelial lining the alveoli and of cells in the connective tissue of cows #4019 and 1039 to the abcam antibody; G-H) Immunoreactivity of mammary tissue of cows #4019 and 1039 to the to the C-term and antibody. Scale bars indicate 25 µm.

We performed IHC studies with antibodies to IL-17A in order to gain information on the relative number and localization of IL-17A-producing cells. In ovalbumin-infused glands of responder cows, a small number of IL-17A-positive cells were found in the connective tissue, most of them in sub-epithelial position or closely associated with the epithelium lining (Figure 8A, B, E–H). Importantly, a similar labeling was obtained with the three different antibodies directed to both ends and the central part of the IL-17A molecule (Figure 8A, B, E–H), thus excluding the possibility of a cross-reaction with another protein, or the reaction with a truncated form of IL-17A. Moreover, the three antibodies cover each of the three exons of the bovine *Il17a* gene, which rules out the possibility of an alternative splicing isoform. Some of the leukocytes that had migrated into the alveoli lumen, including certain neutrophils, were also immunoreactive. An unexpected finding was the strong labeling of the epithelium lining the alveoli. Again, all the three antibodies used reacted with the epithelium lining. Even the antibody against the whole IL-17A (Kingfisher), which did not prove to be well adapted to IHC purpose, faintly labeled the epithelial lining of ASR-reacting MG (results not shown). Clearly, the cytoplasm of epithelial cells of the alveolar monolayer was immunoreactive. Tissue samples from glands of several cows gave the same results (Figure 8A, B, E–H). The N-term antibodies, which yielded the most intense labeling, strongly labeled cells in the parenchyma, the epithelium and a proportion of the cells that had migrated to the lumen of the alveoli (Figure 8A–B). The target peptide completely inhibited the staining of N-term antibody-reactive cells (Figure 8C), demonstrating that the reaction was of the antigen-antibody type and not an unspecific adsorption to the tissue. There was no labeling by rabbit antibodies to ovalbumin in tissue sections revealed with the same conjugated antibody as used with anti-IL-17A antibodies (Figure 8D).

**Figure 9:**
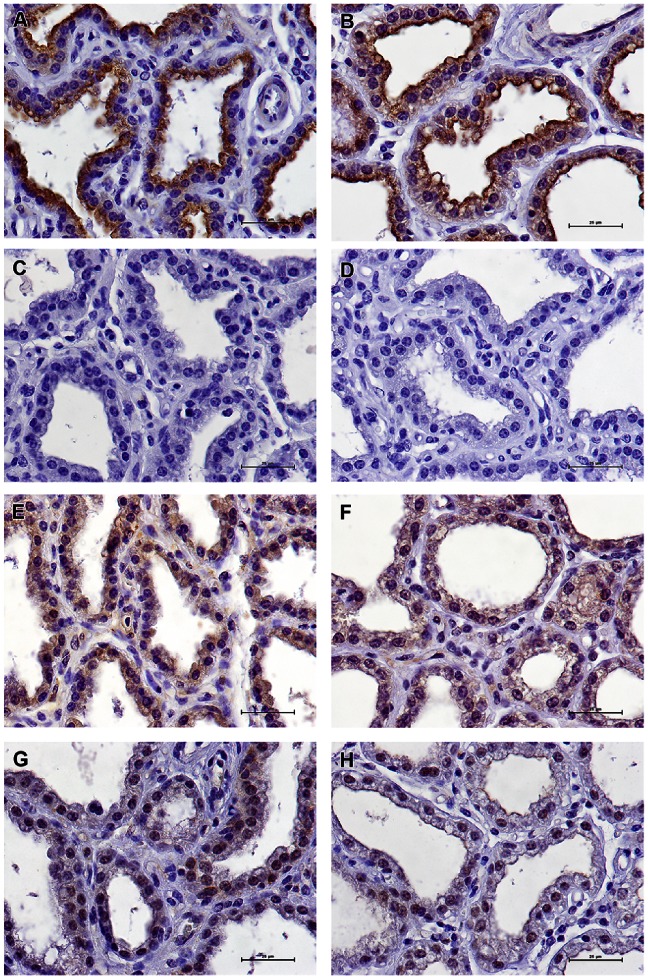
Analysis by immunohistochemistry of representative tissue sections of uninfused, healthy mammary glands. A–B) Immunoreactivity of the apical side of the epithelial cells lining the alveoli to the N-term antibody to IL-17A of cows #1018 and 2014, respectively; C) Inhibition of labeling by Ab to N-terminal IL-17A peptide with the peptide antigen (cow #1018); D) Negative control with Ab to ovalbumin and second antibody conjugated to horseradish peroxidase (cow #1018); E–F) Immunoreactivity of the epithelial lining the alveoli to abcam antibody (cows #1018 and 2014, respectively); G–H) Immunoreactivity of mammary tissue of healthy uninfused quarters of cows 1018 and 2014 to the C-term antibody, respectively. Scale bars indicate 25 µm.

The immunoreactivity of mammary tissue from healthy, uninflamed glands was also investigated. Unexpectedly, a definite labeling of the apical part of the epithelial cells lining the alveoli was obtained with N-term antibodies (Figure 9A–B). This labeling was completely abrogated by the immunizing peptide (Figure 9C), and was not obtained with antibodies to ovalbumin (Figure 9D). Similar labeling was obtained with antibodies directed to the C-terminal or central parts of IL-17A (Figure 9E, G). Mammary tissue from other cows yielded the same immunoreactivity (Figure 9F–H, and results not shown), with the three different antibodies. Of note, contrasting with the labeling of the epithelium of challenged inflamed glands, the labeling of the epithelium of control glands and control cows was limited to the apical side of the cytoplasm and the luminal edge of the epithelium lining.

## Discussion

This investigation was devised to characterize the mammary antigen-specific inflammation induced in cows by intraluminal infusion of a sensitizing antigen. The main objective of this study was to investigate whether cytokines associated with T-cell mediated hypersensitivity were expressed in the MG during an antigen-specific inflammatory response. The results are compatible with the involvement in the reaction of cytokines produced by Th17 and Th1 lymphocytes.

Immunization with ovalbumin led to clinical mastitis and marked milk leukocytosis in sensitized cows upon challenge with the antigen. An interesting observation was the clear-cut response of high-responder and low-responder cows, and the absence of intermediary responsive animals. Nevertheless, all the animals developed antibodies to ovalbumin, although low-responders less than high responders (Figure 4B). We speculated that lymphocytes of the Th17 lineage would contribute to mammary ASR. Accordingly, we searched for the cytokine signature of Th17 cells, and we included IFN-γ as an indicator of Th1 involvement. Th2 cytokines were not considered in the study because eosinophils are not recruited in milk and mammary tissue during mammary ASR elicited by ovalbumin or bacterial antigens [5], [6], a feature that has been verified in the present study (Figure 3). A major finding of our study was the detection of IL-17A in milk and the cytokine profile corresponding to Th17 cells both in milk and mammary tissue, during the early phase of ASR. These findings are quite compatible with our working hypothesis, although they do not constitute the proof that IL-17A, IL-17F and IL-22 were the main cause of the observed inflammation, and that Th17 cells were the main producers of these cytokines. A circumstantial evidence of IL-17A involvement was the chemokine and self-defense gene expression profiles found in milk and mammary tissue, which correspond to the expected response of stromal cells (epithelial and fibroblastic) to IL-17A [27]. More specifically, we have shown previously that bovine MEC respond to IL-17A and IL-17F by upregulating the expression of genes such as *Ccl20*, *TAP* and *Saa3* [16].

A major finding of this study was the detection of IL-17A and IFN-γ in the milk of the challenged MG. To our knowledge it is the first time that IL-17A has been found in milk at the protein level. Several cell types have been reported to produce IL-17A. Production of IL-17A has been shown previously by leukocytes such as CD4+, CD8+ αβ T lymphocytes, γδ T lymphocytes, macrophages and neutrophils [28]–[32]. All these cell types are known to be recruited during mastitis in the bovine MG, but it remains to document which leukocytes are able to produce IL-17A in the bovine species. In our model of antigen-specific inflammation, CD4+ T lymphocytes (Th17) are possible candidates for IL-17A production. IL-17A and IL-17F are not the only cytokines released by Th17 cells. The cytokines IL-22 and IL-26 are primarily produced by activated T cells such as Th17 lymphocytes, and like IL-17A and IL-17F, their primary targets are non-hematopoietic cells such as keratinocytes and other epithelial cells [33], [34]. Transcripts of the *Il22* and *IL26* were found in milk cells and mammary tissue during ASR. Of note, the kinetics of *Il26* expression differed from that of the other genes encoding the Th17-associated cytokines IL-17A, IL-17F and IL-22 (Figure 6), suggesting that IL-26 was produced by different cells or at least that its expression was differently regulated. The capacity of mammary epithelial cells to react to IL-22 and IL-26 and the nature of the response remain to be determined. If the biological activities of IL-26 are still poorly known, activities of IL-22, including epithelial repair [34], are of great potential in the setting of the infected mammary gland. A new finding of our study was the strong expression of *Il21* in milk cells and mammary tissue. IL-21, which is mainly produced by activated CD4+ T cells and NKT cells, has pleiotropic effects on a variety of cell types including B cells, T cells, NK and NKT cells, dendritic cells, monocytes/macrophages and epithelial cells [35]. IL-21 is especially produced in high amounts by Th17 cells, and is considered as the major autocrine cytokine for the differentiation and proliferation of these cells [36]. At the effector end, IL-21 has been shown to be an important player in skin and gut diseases [36], [37]. For example, IL-21 promotes the secretion of CCL20 by gut epithelial cells [38]. As the IL-21 receptor is expressed by a variety of epithelial cells and fibroblasts, it would be important to know whether it is expressed in the mammary tissue and whether MEC respond to IL-21. Overall, the cytokine profile found in milk leukocytes and mammary tissue of challenged glands corresponded to the anticipated Th17 signature.

Another feature of the mammary ASR was the release of IFN-γ in milk and overexpression of *Ifng* in milk cells and mammary tissue. IFN-γ is considered a hallmark cytokine of Th1-mediated immune responses [39]. Th1 cells produce IFN-γ, mainly in response to IL-12 and/or IL-18, which in general induce a massive release of TNF-α, an important mediator of inflammation-related tissue damage. For example it has been shown that TNF-α cooperates with IL-21 to induce the production of metalloproteases by fibroblasts, thus contributing to gut damage [37]. It is thus important to note that TNF-α was not detected in milk during ASR in the context of an intense milk leukocytosis. This contrasts with the high concentrations of TNF-α found in milk in the course of mastitis caused by Gram-negative bacteria, which are characterized by a marked loss of milk production [40], [41]. Our study did not allow the identification of the cell source of IFN-γ. Apart from Th1 cells, several cell types such as mast cells, NK cells or CD8+ T cells are also major producers of this cytokine. Interestingly, certain subsets of Th17 are able to produce IFN-γ, in particular human Th17 cells [42], [43].

In accordance with our hypothesis of Th17 cell involvement in mammary ASR, we attempted to drive the immune response toward a Th17 response by adding MDP and LTA to IFA since these additives are reportedly efficient in humans and mice, with a view to increasing the magnitude of ASR compared to the response obtained with plain IFA as adjuvant. It appeared that MDP and LTA did not enhance the hypersensitivity to ovalbumin. This is at variance with the enhanced effect of complete Freund adjuvant compared to IFA to induce mammary ASR in guinea pigs [7], but in line with the absence of enhancement in cows [5]. The simplest explanation is that the response of cows to MAMPs as adjuvants differs from the response that can be induced in rodents or humans, or that IFA on its own is a potent adjuvant for ASR in the bovine species. This is another indication that adjuvant formulations optimized for mice are not necessarily so for cattle [44].

The overall picture of ASR was a neutrophilic inflammation, particularly during the early phase of the immune response. Neutrophil-targeted chemoattractants were found in milk and their concentrations increased during the reaction (Figure 5). The complement fragment C5a can be found early in mastitic milk [45]. The chemokine CXCL8 (IL-8) was hardly detected in milk before ASR, whereas CXCL3 was already present at high concentration, as expected [23]. The concentrations of these two neutrophil-attracting chemokines increased with time (Figure 5), but their contribution to the onset of neutrophilic inflammation is disputable. The case for milk CXCL8 is particularly dubious, as its concentration was hardly augmented at 8 hpi when a substantial increase in milk neutrophil concentration has already occurred, and the correlation between CXCL8 and concentration and milk leukocytosis was low (Table 2). It has been shown that infusion of recombinant CXCL8 in the lumen of the ovine or bovine mammary gland does not induce milk neutrophilia [46], [47]. Moreover, CXCL8 concentrations (300 pg/ml or less) were dwarfed by CXCL3 concentrations (300 ng/ml). On the other hand, the correlation between CXCL3 concentration and milk leukocytosis was rather high at the beginning of ASR, and it can be speculated that the initial high CXCL3 milk concentration has contributed to the transepithelial migration of neutrophils. Interestingly, the best correlations between immune mediators and cell concentrations in milk were with IFN-γ and IL-17A (Table 2). Nevertheless, chemokines and cytokines in milk may be indicators of inflammatory response rather than effectors of the observed cell recruitment, e.g. milk CXCL8 may reflect the production by milk leukocytes and leakage from mammary tissue, without being the main cause of neutrophil migration into milk. In any case, the initial event leading to milk leukocytosis takes place within the mammary tissue and involves the vascular compartment. In our study we found an overexpression of *Cxcl10* in mammary tissue after challenge. The chemokine CXCL10, also known as interferon-inducible protein-10 (IP-10), targets different cell types including activated lymphocytes and natural killer cells [48]. In particular, it can attract Th1 cells and CD8+ cytotoxic lymphocytes, but also Th17 cells to inflammatory sites [49]. Moreover, it has been shown to increase the recruitment of neutrophils and their phagocytic activity [50]. *Cxcl10* overexpression has been found in mammary tissue or leukocytes attracted in milk after intramammary challenge with *E. coli* or *S. aureus* [51], [52]. As its initial name (IP-10) indicates, *Cxcl10* is induced by IFN-γ, and consequently its overexpression during ASR is in keeping with the expression of *Ifng* in mammary tissue and milk cells. This observation applies also to *Nos2*, which is an interferon-inducible gene [53]. We recently showed that *Nos2* is overexpressed by bovine MEC exposed to IL-17A, in particular in combination with bacterial MAMPs [16]. Other conditions have been associated with the overexpression of *Nos2* in the bovine MG, such as exposure to *E. coli* [54].

In addition to CXCL10, other chemokines that have the potential to attract mononuclear leukocytes were overexpressed in mammary tissue during ASR. The genes encoding the chemokines CCL2 and CCL5 depend on IFN for full expression [55], [56]. CCL5 has been found in milk leukocytes or MEC after stimulation with *E. coli* or LPS but not with *S. aureus* or LTA [57], [58]. This is in keeping with the marked overexpression of *Ccl5* found in our study, in relation with the production of IFN-γ, but probably not of IL-17A which has been reported to repress *Ccl5* expression [59]. The chemokine CCL20 attracts cells that express the cognate receptor CCR6, such as immature dendritic cells, Th17 and regulatory T cells [60]. CCL20 is produced mainly by epithelial cells, but can also be produced by Th17 lymphocytes [61]. We have shown that *Ccl20* is expressed by bovine MEC in response to IL-17A and IL-17F [16], and *Ccl20* has been shown by several groups to be a major response gene of bovine MEC exposed to MAMPs or mastitis-associated bacteria [25], [54], [62]. This strongly suggests that this chemokine plays a major role in directing the recruitment of specific leukocyte subsets in the MG during infection.

A restriction of our study was the limited identification of the cells that produced IL-17A. We had planned to analyze the cells recruited in milk by flow cytometry, but technical problems precluded us doing so. We previously reported that T lymphocytes are recruited in milk during mammary ASR, and that CD8+ cells seemed to precede CD4+ cells by a few hours [63]. Further experiments will be necessary to identify IL-17A producing cells in milk. Interestingly, the Th17 and Th1 transcriptomic profile of milk cells in the early phase of the ASR suggests that milk lymphocytes may be a useful source of cells for further studies to specify their phenotype and functional abilities. On the other hand, there was no coincidence between the peak of IL-17 concentrations in milk and the peak expression of *Il17a* and *Il17f* in milk leukocytes (Figure 5 and 6). This suggests that milk leukocytes were not the main source of milk IL-17, supposing protein secretion was proportional to transcripts numbers, which has not been tested in our study. Alternatively, mammary tissue may be a major source of the cytokines found in milk. Accordingly, we found *Il17a* and *Il17f* genes overexpression in mammary tissue (Figure 7).

We performed IHC studies with antibodies to IL-17A to gain information on the relative number and localization of IL-17A-producing cells. Small numbers of IL-17A-positive cells were found in the connective tissue, in sub-epithelial positions or more closely associated with the epithelium lining (Figure 8). Among these cells are those corresponding to the Th17 cytokine profiles found in the mammary tissue (Figure 7). Immunoreactive cells were also found among migrated cells in the lumen of the alveoli of challenged glands, and it is possible that neutrophils, macrophages and subsets of T lymphocytes were among these immunolabeled cells. Further studies will be necessary to delineate the IL-17-producing cells in the bovine species. An unexpected finding was the strong labeling of MEC by antibodies to IL-17A (Figures 8 & 9). There was a clear difference between ovalbumin-infused and uninfused MG: in challenged quarters, the 3 anti-peptide antibodies used labeled the whole cytoplasm of alveolar epithelial cells, whereas in control quarters only the apical half of the epithelial cells was labeled. Compared to the control quarters, many cells in the connective tissue or subepithelial location along with cells within the alveolar lumen of inflamed glands were labeled in addition to epithelial cells (Figures 8 & 9). Thus there was clearly a qualitative and quantitative difference in IL-17A immunoreactivity consecutively to mammary ASR. Others have reported IL-17-immunoreactive epithelial cells in the lower airways of cystic fibrosis or COPD patients [30], [64], in salivary epithelial cells in Sjögren syndrome [65] and tubular epithelial cells in renal transplants of recipients with graft rejection [66] by using IHC. Further studies will be necessary to investigate the intriguing possibility that MEC are not only targets of IL-17A but also a source of this cytokine and contribute to its secretion in milk during ASR. Even more surprising was the immunoreactivity of the alveolar epithelium of uninflamed glands with antibodies to IL-17A. The labeling was restricted to the apical half of MEC. Yet, IL-17A was not detectable in milk of uninflamed normal glands by ELISA. One possible explanation would be that the concentration of secreted IL-17A was below the lower limit of detection of the ELISA. Epithelial expression of IL-17F in vivo has been described using mRNA analysis and IHC [67], [68], although production of IL-17F in vitro has not been reported [69].

In conclusion, we showed that mammary ASR, which manifested itself by an intense milk leukocytosis, was characterized by the production of IL-17A and IFN-γ but the absence of detectable TNF-α, with a low and inconsistent presence of IL-1β and IL-6. The present study reveals that Th1 and Th17-associated cytokines were induced in the MG during ASR. An unexpected and intriguing finding was the MEC immunoreactivity with antibodies to IL-17A, suggesting that these cells are able to produce IL-17A. Overall, the results of this study raise the possibility that IL-17A and Th17 cells contribute or even are major players in the antigen-specific MG inflammation. Owing to the particular cytokine profile induced, one major implication is that ASR has the potential to substantially modify the magnitude and the nature of the initial response of the MG to bacterial invasion, with a possible major impact on the outcome of infection. Further studies are necessary to identify and characterize the cells producing IL-17 in the MG during ASR, and to assess the consequence of ASR for the defense of the MG against bacterial infections.
